# RON and RONΔ160 promote gastric cancer cell proliferation, migration, and adaption to hypoxia via interaction with β-catenin

**DOI:** 10.18632/aging.101945

**Published:** 2019-05-13

**Authors:** Donghui Zhou, Ling Huang, Yong Zhou, Tao Wei, Lina Yang, Chao Li

**Affiliations:** 1 Department of Oncology, the First Affiliated Hospital, Zhejiang University School of Medicine, Hangzhou 310003, Zhejiang, China; 2 Department of Oncology, the Affiliated Dongnan Hospital of Xiamen University, Zhangzhou 363000, Fujian, China; 3 Department of Medical Oncology, Affiliated Hospital of Inner Mongolia Medical University, Huhhot 010030, Inner Mongolia, China

**Keywords:** gastric cancer, recepteur d’origine nantais, RONΔ160, β-catenin, hypoxia, aging, senescence

## Abstract

Aberrant accumulation of the receptor tyrosine kinase recepteur d’origine nantais (RON) has been verified in gastric adenocarcinoma. Upregulation of RON and its splice variant RONΔ160 contribute to the growth and migration in gastric cancer cells *in vitro*. However, the mechanisms of RON/RONΔ160-mediated gastric cancer growth and metastasis remain vague. We therefore examined the actions of RON, RONΔ160, and β-catenin in gastric cancer cells and tissue samples, and their effects on cell growth *in vitro* and *in vivo*. We found that in gastric cancer samples and cell lines, there was positive correlation between RON/RONΔ160 and β-catenin levels, and that they formed a RON/RONΔ160-β-catenin complex which was translocated to the nucleus. Hypoxia led the binding of hypoxia-inducible factor-1α to the RON/RONΔ160-β-catenin complex, which increased nuclear translocation and expression of downstream oncogenic signaling molecules. Overexpression of RON/RONΔ160 promoted the proliferation and migration of gastric cancer cells, which were also enhanced by hypoxia. Suppression of RON using siRNA or anti‑RON monoclonal antibody diminished gastric cancer cell and tumor growth *in vitro* and *in vivo*. These findings establish a link between the receptor tyrosine kinase RON and β-catenin and provide insight into the mechanism by which they contribute to gastric cancer progression.

## Introduction

Receptor tyrosine kinases (RTKs) are high-affinity cell surface receptors that participate in various signaling pathways regulating cellular proliferation, survival, apoptosis, and migration [1,2]. RTKs also play essential roles in the development and progression in most types of cancer [3]. Indeed, RTK activity is significantly higher in most malignant cancers than in benign tumors or normal tissue, which suggests RTKs may be useful targets for cancer therapy [4–6].

Recepteur d'Origine Nantais (RON), also known as Macrophage-stimulating protein receptor (MST1R), is a member of the c-Met RTK superfamily [7]. RON is reported to be highly expressed in epithelial cancers, including breast, colon, and gastric cancer [8,9], and its expression is always accompanied by generation of various splice variants [10]. So far, nine RON variants have been identified in various primary carcinomas and cancer cell lines, including RONΔ170, RONΔ165, RONΔ165.e11p, RONΔ160, RONE5/6in, RONΔ155, RONΔp110, RONΔ85 and RONΔ55 [11–14]. RON and its splice variants exhibit prominent effects on the occurrence, progression, and metastasis of rectal cancer [15]. In gastric carcinoma, expression of RON and RONΔ165 is significantly up-regulated in the gastric cancer tissue [16]. The truncation of RON to produce RONΔ160 leads to structural changes in the protein that enhances human colorectal adenocarcinomas cells growth *in vitro* and *in vivo* [17,18]. Moreover, forced expression of RONΔ160 effectively enhances the growth and migration of gastric cells *in vitro* and *in vivo* [19]. We therefore hypothesize that RON and RONΔ160 contribute to the pathogenesis of gastric cancer and have an essential stimulatory effect on the proliferation and metastasis of gastric cancer cells.

Once activated by its ligand macrophage-stimulating protein (MSP, MST1), RON initiates multiple downstream signaling cascades that affect cell adhesion and motility, growth, and survival [20,21]. These include the oncogenic RAS/ERK, phosphatidylinostiol-3 kinase (PI3K)/Akt, Wnt/β-catenin, nuclear factor-κB (NF-κB), and focal adhesion kinase (FAK) pathways [22,23]. Among the components of these pathways, β-catenin is a key transcription factor and is required for RON-induced cancer cell outgrowth and migration [24]. Overexpression of RON leads to β-catenin tyrosine phosphorylation and accumulation and the constitutive activation of transcription factor (TCF), which leads to increased expression of the c-Myc and Cyclin D1 oncogenes [25,26]. Knocking down RON using specific siRNA or inhibition of RON’s tyrosine kinase activity with the tyrosine kinase inhibitor BMS-777607 suppresses cell proliferation and metastasis, and extends survival through effects on multiple signaling pathways, especially the β-catenin pathway [27,28]. Although the involvement of RON and RONΔ160 gastric cancer cell growth has been confirmed, it remains unclear whether the effect is mediated by β-catenin signaling. Hypoxia is a hallmark of solid tumors, driving metastatic progression, drug resistance, and recurrence [29]. Hypoxia can promote nuclear translocation of RON and up-regulation of c-Jun, leading to cell outgrowth and hypoxic adaptation [30]. Similarly, hypoxia represses β-catenin-T-cell factor-4 (TCF-4) complex formation, but induces the interaction between β-catenin and HIF-1α, thereby enhancing expression of c-Myc, Cyclin D1, and c-Jun, and cellular adaptation to hypoxia [31]. In that context, we suggest β-catenin is obligatory for RON- and RONΔ160-mediated gastric cancer cell proliferation, and the interaction of RON and β-catenin in gastric cancer cells is more pronounced under hypoxic conditions. To test that idea, we examined in detail the actions of RON, RONΔ160, and β-catenin in gastric cancer cells and their effects on cell growth under normoxic and hypoxic conditions *in vitro* and *in vivo*.

## RESULTS

### RON/RONΔ160 levels correlate positively with β-catenin levels in human gastric cancer tissues

**Figure 1:**
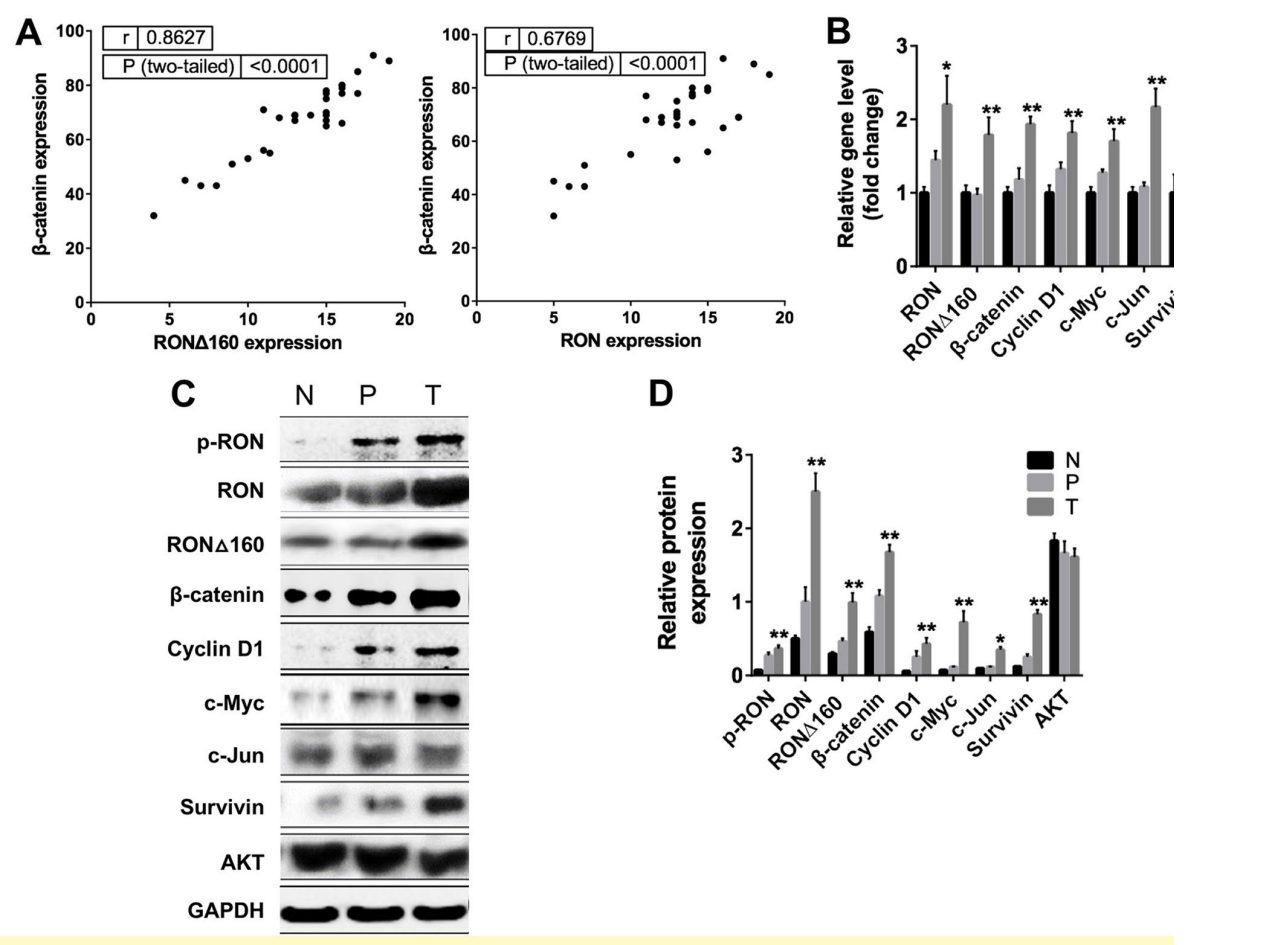
**Correlation between RON and β-catenin in primary human gastric cancer tissues.** (**A**) Scatter plot showing the correlation between RON/RONΔ160 and β-catenin levels in samples of human gastric cancer, paracancerous tissue and normal gastric tissue. (**B**) Relative expression of Wnt/β-catenin signaling pathways related genes in T, P and N were detected by qRT-PCR and normalized to GAPDH. (**C**) Representative Western blotting data of RON and Wnt signaling molecules in T, P and N. (**D**) Quantification of RON and Wnt signaling proteins in T, P and N. *, p<0.05; **, p<0.01 vs N. N: normal gastric tissue. P: paracancerous tissue; T: gastric cancer tissue.

Previous studies indicate that RON is highly expressed in gastric cancer tissues [32], but levels of its RONΔ160 variant and downstream signals are poorly understood. We therefore evaluated the correlation between levels of RON/RONΔ160 and β-catenin (the main mediator of activated RON signaling). By immunoblotting 30 samples of gastric cancer tissue, adjacent paracancerous tissues, and normal gastric tissues, we found that expression of RON and RONΔ160 is positively associated with the level β-catenin protein in gastric cancer samples but not paracancerous or normal tissues (Figure 1A). We also detected a positive linear correlation between the levels of the RON/RONΔ160 and β-catenin transcripts in all three types of gastric tissue studied. Expression of mediators downstream of RON, including c-Myc, Cyclin D1, c-Jun, survivin and AKT, were also increased in gastric cancer tissues (Figure 1B). In addition, levels of c-Myc, Cyclin D1, survivin and c-Jun proteins were increased with increases in RON/RONΔ160 and β-catenin expression in cancer tissues (Figure 1C and 1D). These results show that there is a link between RON/RONΔ160 and β-catenin in gastric cancer tissues, and that β-catenin may be a key regulator of RON signaling during the pathogenesis of gastric cancer.

### RON/RONΔ160 activates Wnt/β-catenin pathway in gastric cancer cells by binding β‑catenin

**Figure 2:**
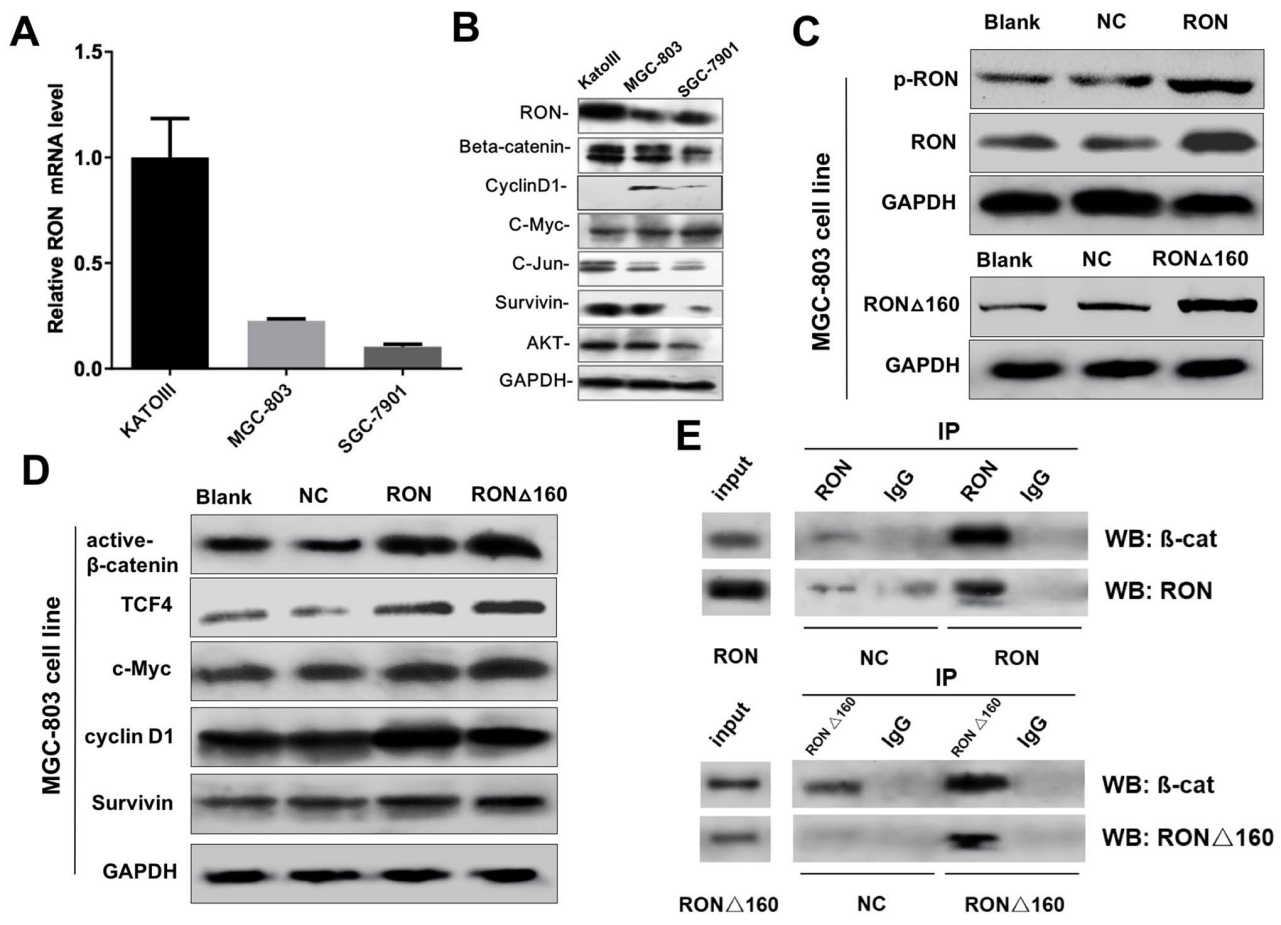
**Effects of RON/RONΔ160 on the activation of β-catenin signaling in human gastric cancer cell lines.** (**A**) Relative mRNA levels of RON and β-catenin signaling-related genes detected in human gastric cancer cell lines using qPCR. (**B**) Western blots showing RON, β-catenin, TCF4, c-Myc, Cyclin D1, and survivin in three gastric cancer cell lines. (**C**) Transfection efficiency of RON and RONΔ160 in MGC-803 cells. (**D**) Expression of β-catenin signaling-related genes after transfection of RON or RONΔ160 into MGC-803 cells. (**E**) Interaction of RON/RONΔ160 and β-catenin in MGC-803 cells determined using co‑immunoprecipitation assays. ** p<0.01 vs Blank group.

To explore the interaction of RON/RONΔ160 and β-catenin at a cellular level, we analyzed the transcript and protein levels of RON and β-catenin and the downstream effector molecules in two gastric cancer cell lines and one normal human gastric mucosal epithelial cells. As in gastric cancer tissues, RON and β-catenin were highly expressed in KATOIII cells. This was accompanied by up-regulation of c-Jun, survivin and AKT, and the downregulation of c-Myc and Cyclin D1. In MGC-803 and SGC-7901 cells, which express lower levels of RON, expression of downstream effectors was also reduced (Figure 2A and 2B). Notably, forcing expression of RON and RONΔ160 by transfecting MGC-803 cells the recombinant plasmids pcDNA3.1-RON and pcDNA3.1-RONΔ160 (Figure 2C) dramatically increased expression of β-catenin, TCF4 and c-Myc, particularly in the RONΔ160 transfectants (Figure 2D). On the other hand, Cyclin D1 expression was enhanced in RON-transfected cells but not those transfected with RONΔ160 (Figure 2D). Both RON and RONΔ160 also increased expression of the anti-apoptotic protein survivin (Figure 2D). In addition, upregulation of RON and RONΔ160 dramatically increased formation of a RON/RONΔ160-β-catenin complex in MGC-803 cells (Figure 2E), suggesting RON is able to recruit β-catenin to trigger downstream signaling cascades.

### Binding of RON/RONΔ160 to β-catenin promotes its nuclear translocation

**Figure 3:**
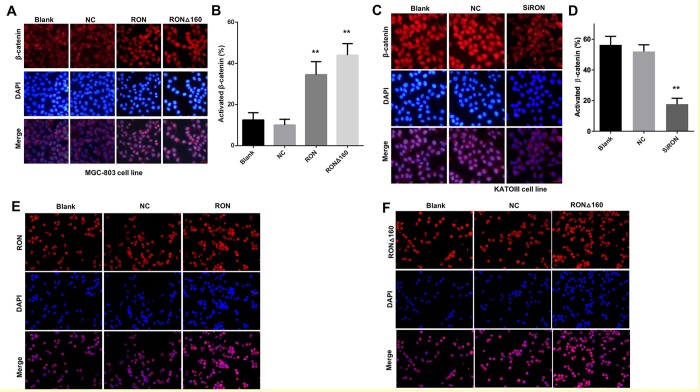
**Role of RON/RONΔ160 in the activation and nuclear translocation of β-catenin.** (**A**) MGC-803 cells were transfected with RON and RONΔ160 and immunostained for RON and β-catenin. (**B**) Nuclear translocation ratio of β-catenin in different groups of MGC-803 cells. (**C**) Immunostaining for RON and β-catenin in KATOIII cells transfected with siRNA targeting RON. (**D**) Nuclear translocation ratio of β-catenin in different groups of KATOIII cells. (**E**) and (**F**) MGC-803 cells were transfected with RON or RONΔ160 and immunostained for RON or RONΔ160. ** p<0.01 vs Blank group. Scale bar = 50 μm in all panels.

Activated β-catenin enters the nucleus and influences the expression of numerous transcription factors and, in turn, multiple cellular progresses [33]. In the present study, RON/RONΔ160 transfection increased the MGC‑803 cell fraction positive for β-catenin and enhanced nuclear translocation of the RON/RONΔ160-β-catenin complex (Figure 3A and 3B), which suggests transfection of RON, or especially RONΔ160, significantly stimulated the expression and nuclear translocation of β-catenin. Conversely, knocking down RON in KATOIII cells suppressed the β-catenin-positive cells fraction and the nuclear levels of activated β-catenin (Figure 3C and 3D). Moreover, Immunofluorescent staining showed that both RON and RONΔ160 translocated into the nucleus in RON/RONΔ160-transfected MGC‑803 cells (Figure 3E and 3F). Thus, interaction between RON/RONΔ160 and β-catenin facilitated nuclear translocation of the RON/RONΔ160-β-catenin complex, which potentially integrates additional nuclear signal transduction.

### Hypoxia enhances binding of HIF-1α to the RON/RONΔ160-β-catenin complex and activation of downstream genes

**Figure 4:**
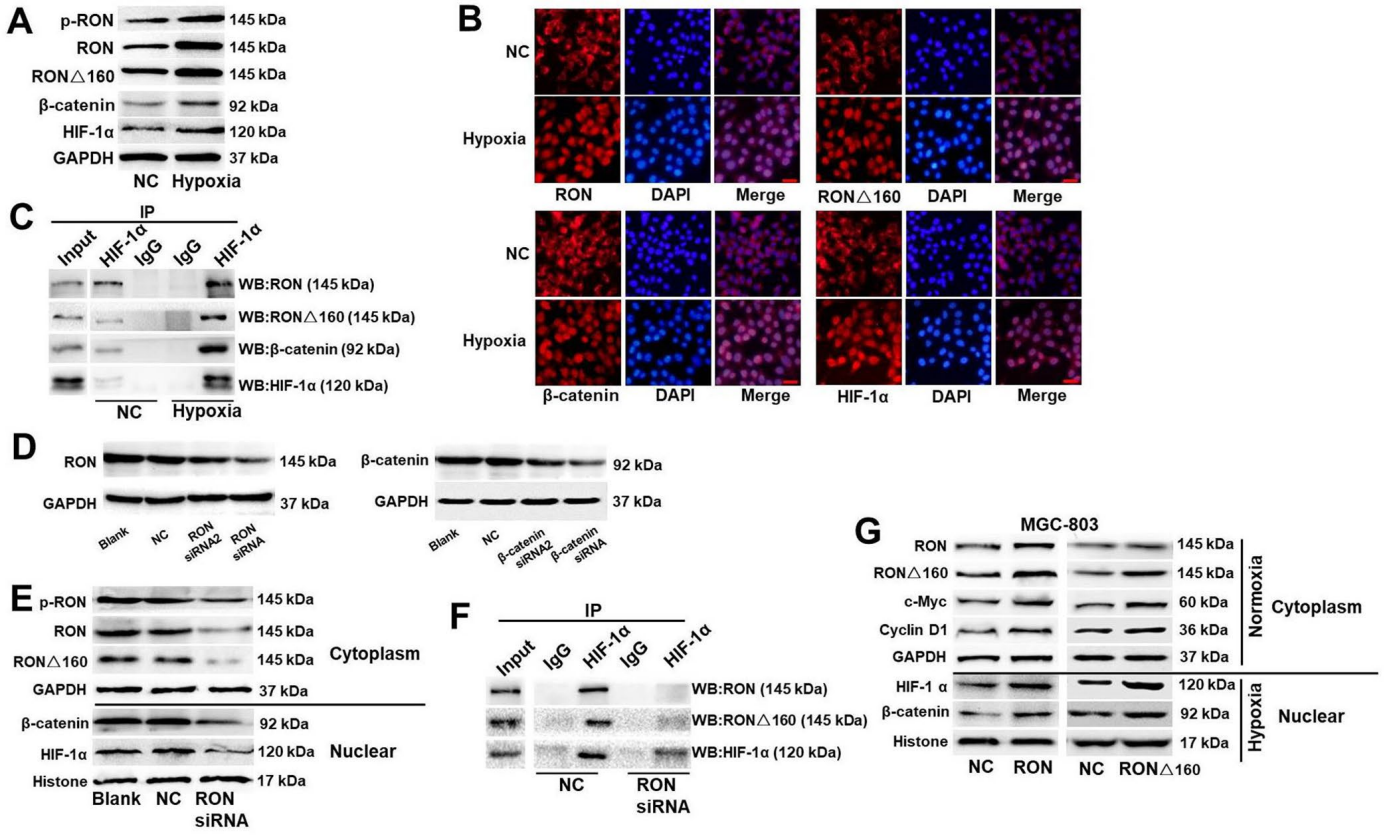
**Effect of hypoxia on HIF-1α binding to the RON/RONΔ160-β-catenin complex.** (**A**) Western blots of p-RON, RON, RONΔ160, β-catenin, and HIF-1α in KATOIII cells under normoxic and hypoxic conditions. (**B**) Immunostaining showing nuclear localization of RON, RONΔ160, β-catenin, and HIF‑1α in KATOIII cells under normoxic and hypoxic conditions. Scale bar = 50 μm. (**C**) Interaction between HIF-1α and the RON/RONΔ160-β-catenin complex in KATOIII cells under normoxic and hypoxic conditions. (**D**) Western blots showing suppression of RON and β-catenin in KATOIII cells transfected with siRNAs targeting RON or β-catenin. (**E**) and (**F**) Western blots (**E**) and co-immunoprecipitation assays (**F**) showing the suppressive effect of RON siRNA the interaction between HIF-1α and the RON/RONΔ160‑β-catenin complex. (**G**) Western blots showing levels of RON, RONΔ160, β‑catenin, and HIF-1α in MGC-803 cells overexpressing RON of RONΔ160. Scale bar = 50 μm in all panels.

Nuclear translocation of RON may occur in serum-starved and hypoxic cancer cells [30,34], and hypoxia can also stimulate nuclear translocation of β-catenin. This suggests RON and β-catenin were involved in hypoxia-inducible signaling networks in gastric cancer cells. Consistent with that idea, cytoplasmic and nuclear levels of p-RON, RON, RONΔ160, β-catenin, and the hypoxia-inducible factor HIF-1α are all increased under hypoxic conditions in KATOIII cells (Figure 4A and 4B). Given the co-localization of RON/RONΔ160, β-catenin, and HIF-1α, we next immunoprecipitated nuclear and cytoplasmic proteins from KATOIII cells to evaluate the relationships among them. We detected a strong interaction among them in the nucleus but not the cytoplasm, and the interaction was stronger under hypoxic conditions (Figure 4C). Next, RON siRNAs and β-catenin siRNAs were used to inhibit the expressions of RON and β-catenin in cells, respectively (Figure 4D). As expected, knocking down RON and β-catenin expression suppressed entry of HIF‑1α into nucleus and binding of HIF-1α to the RON-β-catenin complex (Figure 4E). Conversely, nuclear translocation of β-catenin was obviously increased in MGC-803 cells transfected with RON/RONΔ160, and this was accompanied by increased of c-Myc and Cyclin D1 expression under normoxic conditions (Figure 4F). Under hypoxia, overexpression of RON and RONΔ160 enhanced nuclear β-catenin and HIF-1α levels (Figure 4G).

Hypoxia promotes tumor growth and increases the risk of metastasis. For example, it causes the separation of β-catenin and TCF-4 but promotes the interaction of β-catenin and HIF-1α, while reducing expression of c-Myc and Cyclin D1 [25,26]. In the present study, we observed that hypoxia enhances expression of c-Jun and CA-IX, which are regulated by HIF-1α under hypoxic conditions, and these changes were significantly increased in RONΔ160-transfected cells (Figure 4G). These findings together with the data presented above provides strong evidences that binding of HIF-1α to the RON/RONΔ160-β-catenin complex is essential for the growth of tumor cells under hypoxic conditions.

### β-catenin is required for RON/RONΔ160-mediated gastric cancer cell growth and migration

**Figure 5:**
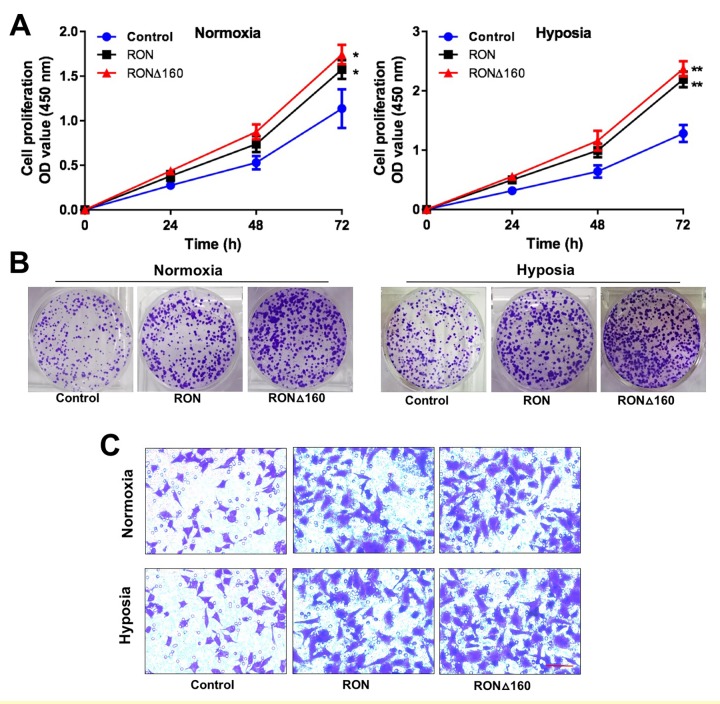
**RON and RONΔ160 promote the proliferation and invasiveness of gastric cancer cells.** (**A**) CCK-8 assays showing growth of RON- and RONΔ160-transfected MGC-803 cells under normoxic and hypoxic condition. (**B**) Colony formation by RON- and RONΔ160-transfected MGC-803 cells under normoxic and hypoxic conditions. (**C**) Transwell assays showing MGC-803 cell migration. (**D**) The quantification of migration cells. Scale bar = 50 μm. ** p<0.01 vs Control group.

**Figure 6:**
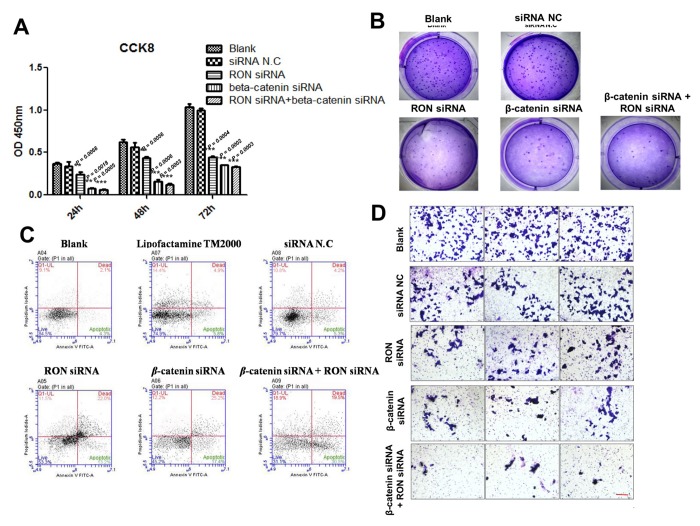
**Impact of RON and β-catenin knockdown on KATOIII cell growth, survival and migration.** (**A**) and (**B**) CCK-8 and colony formation assays showing the effects of RON and/or β-catenin knockdown using targeted siRNAs on KATOIII cell growth. (**C**) Flow cytometric analysis of apoptosis among KATOIII cells transfected with RON and/or β-catenin siRNAs. (**D**) Transwell assays showing effect of RON and/or β-catenin knockdown on KATOIII cell migration. Scale bar = 50 μm. ** p<0.01 vs Blank group.

Because RON/RONΔ160-β-catenin signaling mediates hypoxia-induced signaling, we assessed their effects gastric cancer cell growth under hypoxic conditions. Overexpression of RON or RONΔ160 stimulated MGC-803 cell proliferation and colony formation, and this effect was increased under the condition of hypoxia (Figure 5A and 5B). Upregulation of RON or RONΔ160 stimulated gastric cancer cell migration, and that effect, too, was enhanced by hypoxia (Figure 5C and 5D). On the other hand, RON knockdown strongly suppressed KATOIII cell growth and colony formation efficiency. Interestingly, downregulation of β-catenin in KATOIII cells had an even greater inhibitory effect on cell growth and colony generation, but there was no synergistic effect when RON and β-catenin were simultaneously inhibited (Figure 6A and 6B). This data suggest that β-catenin governs RON/RONΔ160-mediated signal transduction networks in gastric cancer cells. Cell apoptosis and transwell assays revealed that knocking down RON and β-catenin promoted KATO III cell apoptosis and inhibited their migration (Figure 6C and 6D). Thus, β-catenin appears to be the pivotal mediator in the RON/β-catenin signaling pathway, playing a critical role on the gastric cancer cells growth, survival, and metastasis.

### RON/β-catenin signaling is essential for tumor growth *in vivo*

**Figure 7:**
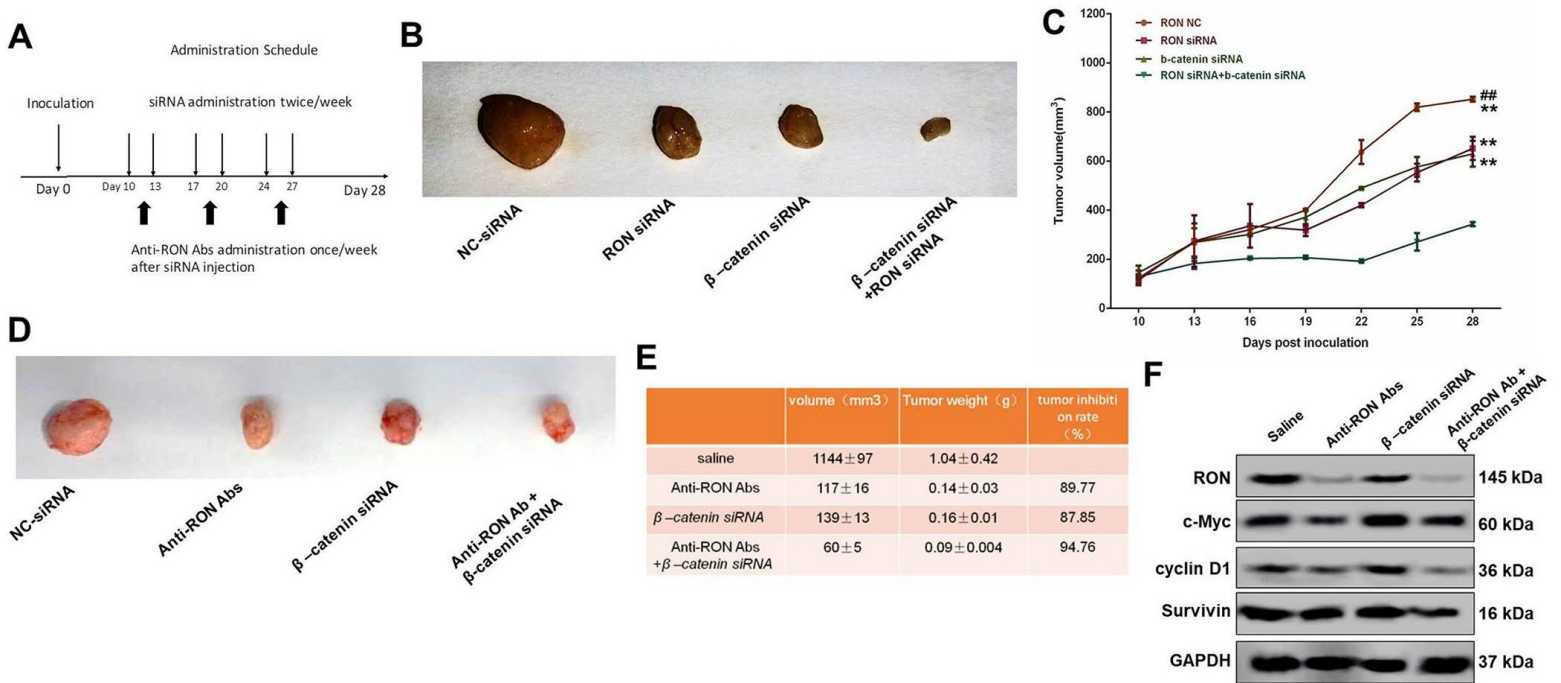
**Effect of RON/β-catenin signaling on tumor growth of KATOIII cells.** (**A**) Flowchart showing the experimental protocol for the xenograft model. (**B**) Xenografted tumors were injected with RON and/or β-catenin siRNAs twice per week. The mice were sacrificed and the tumor was collected 28 days after initial injection. (**C**) Statistical analysis of the tumor growth curves. (**D**) Xenografted tumors were injected with RON siRNAs twice a week and with anti-RON antibodies once a week. The mice were sacrificed and the tumor collected 28 days after initial injection. (**E**) Statistics of tumor volume and weight in the different groups. (**F**) Western blots showing tumoral levels of RON, c-Myc, cyclin D1 and survive proteins. ** p<0.01 vs RON NC; ^##^ p<0.01 vs RON siRNA.

To evaluate the tumor-promoting action of RON/β-catenin signaling on gastric cancer *in vivo*, xenograft experiments were performed. The experimental protocol is shown in Figure 7A. Following subcutaneous injection of KATOIII cells, the tumor size was markedly smaller when the injected cells had been transfected with siRNAs targeting RON or β-catenin, and the smallest tumors were obtained after knocking down both RON and β-catenin (Figure 7B). As shown in Figure 7C and D, the rate of tumor growth was significantly reduced by RON or/and β-catenin knockdown, which was consistent with the *in vitro* data.

RTKs are reportedly key prognostic factors in gastric cancer, and a number of drugs targeting RTKs are currently being used clinically [6]. RON is a novel prognostic biomarker and therapeutic target for gastric adenocarcinoma [35] and may be a useful target in the treatment of gastric cancer. To test that idea, we administered anti-RON antibody to xenografted mice to assess its antitumor effect *in vivo*. We found that in mice receiving the antibody, the tumor volume (117±16 mm^3^ vs 1144 ± 97 mm^3^) and tumor weight (1.04 ± 0.42 mm^3^ vs 0.14 ± 0.03 mm^3^) were notably reduced as compared to mice receiving saline (Figure 7E). The inhibitory effect of the antibody was similar to that of β-catenin knockdown (Tumor volume: 139 ± 13 mm^3^, tumor weight: 0.16 ± 0.01 g) (Figure 7E). Moreover, treatment with both anti-RON antibody and β‑catenin siRNA suppressed tumor growth to an even greater extent (Tumor volume: 60 ± 5 mm^3^, tumor weight: 0.09 ± 0.004 g) (Figure 7E). In addition, administration of an anti-RON antibody also reduced tumoral levels of its downstream targets, including c-Myc, Cyclin D1, survivin, c-Jun, and CA-IX, and the effect was even greater with administration of anti-RON antibody plus β‑catenin siRNA (Figure 7F). These results suggest that RON/β-catenin act as an essential hub of signal transduction mediating progression of gastric cancer, and may be an effective drug target for the treatment of gastric cancer.

## DISCUSSION

Gastric cancer is a major cause of cancer death worldwide [36]. Currently, complete resection is the main therapeutic strategy. And despite recent advances in therapeutic methods, including immunotherapy and receptor tyrosine kinases inhibitors (TKIs), gastric cancer remains one of the most intractable solid tumors characterized by frequent posttreatment relapse and metastasis [37]. There is thus an urgent need to identify novel drug targets with the potential to improve the outcomes of gastric cancer patients.

The receptor tyrosine kinase RON is a member of c-MET proto-oncogene family and a novel prognostic marker and therapeutic target for gastroesophageal adenocarcinoma; 56.1% of gastric carcinomas exhibit high expression of both RON and RONΔ165, leading to a poor survival rate [16,38]. In the present study, we confirmed that both RON and RONΔ160 are significantly upregulated gastric cancer tissues as compared to paracancerous and normal tissues, suggesting RON and its splice variants may act as tumor promoters in gastric adenocarcinoma.

Aberrant β-catenin expression is associated with most types of cancer, including hepatocellular, colorectal, lung, and gastric cancers [39]. Upon activation, β-catenin translocates from the cytoplasm to the nucleus and through interaction with the transcription factor TCF4 triggers expression various genes, including c-Myc, Cyclin D1, and c-Jun [40]. The protein tyrosine kinase MET also complexes with β-catenin to integrate multiple downstream signals contributing to the development and progression of cancer [41]. However, the interactions between RON and/or its splice variants and β-catenin in gastric cancer remains poorly understood. Here we showed that there is a direct interaction between RON/RONΔ160 and β-catenin and that transfecting cells with RON/RONΔ160 leads to increased levels of β‑catenin, constitutive activation of TCF4, and elevated levels of proteins encoded by β‑catenin/TCF4 target oncogenes, such as c-myc and cyclin D1. In addition, Overexpression of RON/RONΔ160 promoted translocation of the RON/RONΔ160-β-catenin complex into the nucleus, while RON/RONΔ160 knockdown suppressed that effect. It is therefore conceivable that the RON/RONΔ160-β-catenin complex potentially initiates activation of downstream targets in the β-catenin signaling pathway in gastric cancer.

Tumor cell microenvironments often induce activation of Wnt/β-catenin signaling and facilitate the adaptation of gastric cancer cells to hypoxia, leading to gastrointestinal tumorigenesis [42,43]. An earlier study showed that the interaction between HIF-1α and β‑catenin contributes to migration of hypoxic gastric cancer cells [44]. We therefore speculated that there is a close relationship between the RON/β-catenin complex and HIF-1α under hypoxic conditions. In the present data, we observed that hypoxia enhanced RON/β-catenin nuclear translocation and the binding of RON/β-catenin to HIF-1α. In addition, Overexpression of RON under hypoxic conditions promoted expression of HIF-1α and vice versa. This suggests RON/β-catenin signaling may be a key mediator in hypoxia-induced elevation of gastric cancer cell proliferation, migration, and survival.

Although RON/RONΔ160 and β-catenin appear to facilitate gastric cancer progression by enhancing the cell proliferation and migration [19,45], it remains unclear whether β-catenin is indispensable for those effects. Our *in vitro* proliferation and migration experiments and *in vivo* xenograft assay showed that suppressing RON using siRNA or an anti-RON monoclonal antibody observably restrained the development and progression of gastric cancer, and that simultaneously knocking down RON and β-catenin had an even greater inhibitory effect. Thus, while β-catenin may not be indispensable, it clearly enhances the pathogenic effect.

In summary, our findings revealed that direct interaction between RON and β-catenin and their contribution to the pathogenesis of gastric cancer and that the variant RONΔ160 exerts similar effects. Moreover, under the hypoxic condition, like those seen in solid tumors, the RON/β-catenin complex binds HIF-1α, which further enhances the tumor growth. We therefore suggest that future investigation of that RON as new therapeutic target for anti-cancer immunotherapy is warranted.

## MATERIALS AND METHODS

### Cells, tissues and mice

A total of 30 samples of gastric cancer tissue (14 cases), paracancerous tissue (11 cases) and normal gastric tissue (5 cases) were collected from First Affiliated Hospital, Zhejiang University School of Medicine. Written consent was obtained from all patients. The recombinant plasmid pcDNA3.1-RONΔ160 and blank plasmid pcDNA3.1 were purchased from Thermo Fisher Scientific (Waltham, MA, USA). Female Balb/c nude mice (5-6 weeks) were purchased from Vital River (Beijing, China) and maintained in a room under controlled light (12 h/day) and temperature (22 ± 2°C) conditions.

### Cell culture and cell treatment

The KATOIII, SGC-7901, and MGC-803 human gastric cancer cell lines were obtained from the American Type Culture Collection (ATCC, Rockville, MD, USA). All gastric cancer cell lines were cultured in RPMI-1640 medium supplemented with 10% fetal bovine serum (Thermo Fisher Scientific, Waltham, MA, USA) at 37°C in a humidified incubator under 5% CO_2_/95% air. For transfection of RON or RONΔ160, cells at 70-80% confluence, and plasmid-transfection reagent mixture was added to the plate for 20 min. Twenty-four hours later, the cells were collected for further investigation. For knockdown assays, siRNAs targeting RON or β-catenin were transfected into cells at 30-50% confluence using LipofectamineTM 2000 regent (Thermo Fisher Scientific, Waltham, MA, USA). All siRNAs were purchased from Thermo Fisher Scientific (Waltham, MA, USA). For hypoxic treatment, the cells were cultured at 1% O_2_ in a modulator incubator.

### CCK-8 assay

Gastric cancer cell proliferation was assessed using a Cell Counting Kit-8 (CCK-8, Dojindo, Kumamoto, Japan). Untreated cells (Blank) and cell transfected with siRNA N.C, RON siRNA, β-catenin siRNA, RON siRNA + β-catenin siRNA, and RON siRNA + β-catenin were plated at a density of 3 x10^3^ cells/well in 96-well plates and grown for 24, 48, or 72 h in complete RPMI-1640 medium. The live cell fractions were then determined using the CCK-8 kit according to the manufacturer’s instructions. The absorbance (OD) at 450 nm was measured using a multiscan plate reader.

### Cell apoptosis assay

Cells were collected using trypsin without EDTA, washed twice with ice-cold PBS, then resuspended in 500 μl of binding buffer containing 5 μl of Annexin V and incubated for 15 min at room temperature in the dark. Five minutes before measurement, 5 μl of propidium iodide (PI) were added into each sample, and the percent apoptotic cells were determined using flow cytometry (BD, Franklin Lake, NJ, USA).

### Transwell assay

Migration assays were performed using 12-well plates with Transwell inserts. Cells were plated at a density of 1x10^5^ cells/chamber in the upper chambers on 8.0-μm, fibronectin-coated, polycarbonate membranes (Millipore, Billerica, MA, USA) and cultured in fresh serum-free medium. The lower chambers contained 600 μl of medium with 10% FBS. After incubation for 24 h, the upper chambers were removed, and the cells fixed for 30 min with ethanol. The cells were then washed twice with PBS and stained with 0.1% crystal violet for 20 min. Cell migrating through the membrane were counted using a microscope.

### Quantitative real-time PCR (qRT-PCR)

Total RNA was isolated from cells and tissues using Trizol Reagent (Roche, Indianapolis, IN, USA) according to manufacturer’s instructions, after which 1 μg of mRNA was used as template for reverse transcription PCR with a First-Strand cDNA Synthesis Kit (Thermo Fisher Scientific, Waltham, MA, USA). The PCR reaction was performed using SYBR Green (Roche, Indianapolis, IN, USA) with the following primers: RON, 5’-CGCGGATCCGGCGCTCTTGGCTGAGGTCAAG-3’ (forward) and 5’-GGAATTCGGCACTATCTGCTCCACCTCCCC-3’ (reverse). β-actin, 5’- CTACAATGAGCTGCGTGTGG-3’ (forward) and 5’- CTACAATGAGCTGCGTGTGG-3’ (reverse). β-actin served as an internal control. Relative gene expression levels were calculated using the 2-∆∆Ct method.

### Western blotting, nuclear and cytoplasmic protein preparation and co‑immunoprecipitation

Total cell lysates were extracted as described previously [32], after which the proteins were separated on 8-12% SDS-PAGE. The protein levels were detected after incubation with specific primary antibodies overnight at 4°C followed by HRP-conjugated secondary antibody for another 1 h at room temperature. The primary antibodies included anti-phospho-RON (p-RON), anti-RON, anti-β-catenin, anti-c-Myc, anti-Cyclin D1, anti-TCF4, anti-HIF-1α, and anti-survivin (Abcam, Cambridge, MA, USA). For nuclear and cytoplasmic protein preparation, nuclear and cytoplasmic proteins were extracted using a NE-PER Nuclear and Cytoplasmic Extraction Kit (Thermo Fisher Scientific, Waltham, MA, USA). Western blotting was then performed to evaluate proteins differentially expressed in nuclear and cytoplasmic. To assess the interaction between RON and β-catenin, cell lysates were extracted using RIPA lysis buffer (Cell Signaling Technology, Danvers, MA, USA) and incubated with anti-RON, anti-RONΔ160, and IgG overnight. The mixture was then incubated with protein A/G beads for 3 h at room temperature. After centrifugation, the precipitated complex was boiled with 2 x SDS loading buffer for 5 min at 95°C, after which Western blotting was performed with the supernatant to assess the interaction between RON and β-catenin. Blot density was then quantified using Image J software.

### Immunofluorescence

In brief, cells were grown on cover glasses for 24 h then washed for twice with ice-cold PBS buffer. After fixing in 3% paraformaldehyde for 30 min at 4°C, the cells were washed with 50 mM NH4Cl and permeabilized with 0.1% Triton100 for 15 min at room temperature. The cells were then incubated with anti-RON, anti-β-catenin or anti-HIF-1α antibodies for 1 h at room temperature and labeled using Texas red-conjugated secondary antibodies. The immunolabeled cells were examined using Carl Zeiss LSM5 EXITER laser scanning confocal microscope. The immunofluorescence quantification for nuclear translocation was performed by manual counting (3 fields per section).

### Xenograft mouse model

Balb/c nude mice were randomly divided into case and control groups and allowed to adapted for 1 week. Thereafter, 2 x 10^6^ gastric cancer cells were subcutaneously injected into the armpit area (5 mice per group). Ten days later, the xenografted mice were intratumorally injected with 50 nM RON or/and β-catenin siRNAs twice/weekly. Another xenografted mice were intratumorally injected with 50 nM β-catenin siRNAs twice/weekly followed by intraperitoneal injection of 20 μg/ml anti-RON antibody (Zt/g4) once/weekly. Tumor size was measured every 3 days. The mice were sacrificed and the tumor was excised 28 days after the initial injection. Tumors were photographed and the homogenized for protein and RNA extraction. Tumor size was calculated using the formula: 0.5 × length × width^2^. Monoclonal antibodies Zt/g4 specific to RON sema domain was kindly supplied by Professor Yao (Laboratory of Cancer Biology and Therapeutics, First Affiliated Hospital, Zhejiang University School of Medicine).

### Statistical analysis

Three independent experiments were performed. All statistical analyses were done using GraphPad Software, Prism 5.0. Numerical data are presented as the mean ± SEM. Values of P<0.05 are considered significant.

### Ethics approval and consent to participate

Ethical approval for the present animal study and the usage of human tissues were received from the Zhejiang University School of Medicine Ethics Committee (Hangzhou, China). National Institutes of Health guide for the care and use of laboratory animals was followed by all authors when carried out the animal study.

### Consent for publication

All patients provided written informed consent for the publication of all associated data in this study.

### Availability of data and materials

The datasets used and/or analyzed during the current study are available from the corresponding author on reasonable request.
